# Detection of Recent HIV-1 Infection Using a New Limiting-Antigen Avidity Assay: Potential for HIV-1 Incidence Estimates and Avidity Maturation Studies

**DOI:** 10.1371/journal.pone.0033328

**Published:** 2012-03-27

**Authors:** Yen T. Duong, Maofeng Qiu, Anindya K. De, Keisha Jackson, Trudy Dobbs, Andrea A. Kim, John N. Nkengasong, Bharat S. Parekh

**Affiliations:** Division of Global HIV/AIDS, Center for Global Health, Centers for Disease Control and Prevention, Atlanta, Georgia, United States of America; Rush University, United States of America

## Abstract

**Background:**

Accurate and reliable laboratory methods are needed for estimation of HIV-1 incidence to identify the high-risk populations and target and monitor prevention efforts. We previously described a single-well limiting-antigen avidity enzyme immunoassay (LAg-Avidity EIA) to detect recent HIV-1 infection.

**Methods:**

We describe here further optimization and characterization of LAg-Avidity EIA, comparing it to the BED assay and a two-well avidity-index (AI) EIA. Specimen sets included longitudinal sera (n = 393), collected from 89 seroconverting individuals from 4 cohorts representing 4 HIV-1 subtypes, and sera from AIDS patients (n = 488) with or without TB co-infections from 3 different cohorts. Ninety seven HIV-1 positive specimens were purchased commercially. The BED assay, LAg-Avidity EIA, AI-EIA and HIV serology were performed, as needed.

**Results:**

Monitoring quality control specimens indicated high reproducibility of the LAg-Avidity EIA with coefficient of variation of <10% in the dynamic range. The LAg-Avidity EIA has an overall mean duration of recency (ω) of 141 days (95% CI 119–160) at normalized optical density (ODn) cutoff of 1.0, with similar ω in different HIV-1 subtypes and populations (132 to 143 days). Antibody avidity kinetics were similar among individuals and subtypes by both the LAg-Avidity EIA and AI-EIA compared to the HIV-IgG levels measured by the BED assay. The false recent rate among individuals with AIDS was 0.2% with the LAg-Avidity EIA, compared to 2.9% with the BED assay. Western blot profiles of specimens with increasing avidity confirm accurate detection of recent HIV-1 infections.

**Conclusions:**

These data demonstrate that the LAg-Avidity EIA is a promising assay with consistent ω in different populations and subtypes. The assay should be very useful for 1) estimating HIV-1 incidence in cross-sectional specimens as part of HIV surveillance, 2) identifying risk factors for recent infections, 3) measuring impact of prevention programs, and 4) studying avidity maturation during vaccine trials.

## Introduction

In the last decade, significant national and international efforts have focused on HIV prevention, care, and treatment of HIV-infected individuals in many countries. Major international initiatives such as the President's Emergency Plan for AIDS Relief (PEPFAR) aims to prevent 12 million new infections. Recent focus on combination prevention is also geared towards reducing new transmissions. Although major strides have been made on several fronts, measuring the impact of these scaled up programs on HIV incidence has remained challenging.

The burden of the HIV epidemic is routinely measured by prevalence, the proportion of individuals with HIV. However, monitoring emerging epidemics in subpopulations, such as those most at risk for infection, are not apparent in these numbers [1]. The measurement of incidence can elucidate transmission dynamics of new HIV infections and allow tracking of epidemiological trends. Additionally, incidence measurements can help target prevention programs and determine the effectiveness of these programs in reducing HIV infections. However, development of a reliable method to estimate HIV-1 incidence has remained elusive [2], [3].

Although prospective follow-up studies and mathematical modeling can be used to derive HIV-1 incidence estimates, there are limitations to these approaches that include: the complexities of following a cohort of individuals at risk for acquiring HIV infection, such as high costs, recruitment bias, and the Hawthorne effect where participants modify their behavior after enrollment in the study, and biases in the assumptions that lead to inaccuracy for modeled-based estimates. Therefore, laboratory-based methods for incidence estimation have continued to be attractive due to the simplicity, ease of testing, low cost and application to single cross-sectional specimens collected during routine surveys. Laboratory assays were developed and applied to detect acute and recent HIV-1 infection for the purpose of estimating HIV-1 incidence starting in the mid-1990s [4], [5], [6], [7], [8], [9], [10], [11], [12]. Diagnosis of acute infection relies on the detection of p24 antigen or RNA prior to elicitation of HIV antibodies. However, acute detection methods are not ideal for incidence estimation because of the short duration of the RNA/p24 detection period and consequent impact of the variability of the “mean acute period” [12]. A change of one to two weeks in the mean acute period could significantly alter the precision of incidence estimates because of the short duration of the acute phase. Moreover, this approach requires testing of a large number of HIV negative individuals, which can be very expensive and is not practical for resource-constrained settings. Since 1998, the focus of laboratory-based methods has been to detect recent seroconversions among HIV-1 antibody positive individuals based on maturation of evolving antibodies that usually follows a predictable pattern. Earlier efforts were focused on development of methods, such as less-sensitive EIAs or the BED-capture EIA (BED-CEIA), to quantify parameters, such as antibody titer [4], [9] or indirect measurements of HIV antibodies levels [6], [13], for the purpose of recent or long-term classification. Currently, the BED assay is the only commercial assay developed specifically for incidence surveillance and is used widely for incidence estimation and characterization of recent infections [14], [15], [16], [17], [18], [19]. Additional studies have demonstrated limitations of these approaches due to HIV-1 subtype variations, as in the case of modified commercial assays [20], [21], or due to population differences, as in the case of BED assay [22]. Therefore, novel and more robust laboratory tools that go beyond current methods are needed to estimate HIV-1 incidence.

Recent efforts around incidence estimation are focused on measurement of antibody avidity, which increases over time following seroconversion. Antibody avidity is believed to be more robust than antibody titer because it is a functional property of maturing antibodies [23]. Most descriptions of avidity measurements utilize commercial HIV diagnostic assays [7], [10], [24] that are modified to measure avidity index. Avidity is commonly expressed as an “avidity index (AI),” a ratio of optical density (OD) values from a two-well assay, where one well is treated with a chaotropic reagent to dissociate low-avidity antibodies and a second untreated well is used as a control. However, commercial assays are likely to have subtype-dependent performance [20], [21]. In addition, commercial diagnostic assays include multiple antigens with several different epitopes that elicit antibodies with varying timeline and maturation kinetics, further complicating avidity measurements [23]. We recently described two novel assays, including a two-well avidity index EIA (AI-EIA) and a single-well limiting antigen avidity EIA (LAg-Avidity EIA), to measure avidity of HIV antibodies [25]. Key features of the one-well assay are: 1) use of rIDR-M, a multi-subtype recombinant protein that covers the immunodominant region (IDR) of gp41 of HIV-1, group M, 2) the limiting amount of antigen available to allow binding of only high-avidity antibodies, and 3) use of pH 3.0 buffer to further assist dissociation of low avidity antibodies. We have now further optimized and characterized the assay; this report describes performance characteristics of the assay in different populations and HIV-1 subtypes, demonstrating that the assay has similar performance among individuals infected with different subtypes and across different populations and may be an important tool for measurement of HIV-1 incidence worldwide.

## Materials and Methods

### Longitudinal specimens from incident infections

Available longitudinal specimens (n = 393) from 89 seroconverting individuals, collected as part of 4 different cohorts from Amsterdam (subtype B, N[# of subjects] = 25, n[# of specimens] = 100), Trinidad [subtype B, N = 7, n = 71], Kenya [subtype A and D, N = 34, n = 81] and Ethiopia [subtype C, N = 23, n = 144], were tested with the three incidence assays (BED assay, LAg-Avidity EIA and AI-EIA). These specimens were obtained from various collaborators and collected as part of different longitudinal cohort studies; they were also used recently to determine the mean duration of recency for the BED assay [22]. The mid-point of last negative date and first positive date was used to infer the time of seroconversion and used to calculate time since seroconversion for subsequent positive specimens.

### Cross-sectional HIV-1 positive specimens

A total of 97 HIV-1 positive plasma specimens were purchased from Boca Biolistics, Inc. (Coconut Creek, FL) and used for the development of controls and calibrator (CAL), optimization of the LAg-Avidity EIA, and as a cross-sectional specimen set for testing. These bulk volume specimens (∼200 mL each) were thoroughly characterized with respect to HIV serology testing and HIV-1 Western blot assay.

### Specimens from individuals with AIDS

Specimens from individuals (N = 488) with AIDS (CD4<200) were derived from three sources and used to estimate a false recent rate for the LAg-Avidity EIA. Two hundred and sixty one specimens (n = 261) were collected in the 1990s from treatment-naïve women with AIDS enrolled in the HIV Epidemiologic Research Study (HERS) [26]. Additional specimens were from Thailand (n = 128) and Cote d'Ivoire (n = 99), collected in the 1990s from treatment-naïve AIDS patients with (Cote d'Ivoire) or without (Thailand) tuberculosis (TB).

### LAg-Avidity EIA

The design and development of the rIDR-M recombinant protein was previously described [25], [27]. A large batch of rIDR-M was commercially made for coating consistency (Proteos, Kalamazoo, MI); results presented here were obtained using plates coated with this large batch of rIDR-M. The protein was dissolved in dimethyl sulfoxide at a high concentration of 5 mg/mL, aliquoted and stored frozen at −80°C. Nunc MaxiSorp 96-well microplates (Thermo Scientific, Rochester, NY) were coated with 0.063 µg/ml of rIDR-M which was determined to be the optimal antigen concentration for the assay. Each well was coated with 100 µL of freshly prepared 0.063 µg/mL rIDR-M in cold 0.1 M phosphate buffer solution (PBS), pH 7.4 containing 0.1% sodium azide (Sigma Aldrich, St. Louis, MO) that was 0.2 µM-filtered prior to use. The plates were incubated overnight at 4°C, then washed two times with 300 µL/well of PBS containing 0.1% Triton X-100 (Sigma Aldrich) (PBS-Triton). Plates were blocked with 200 µL of 5% nonfat dry milk powder (Nestle Carnation, Wilkes-Barre, PA) in PBS-Triton (milk buffer) for 1 hr at 37°C and then washed two times with 300 µL/well of PBS-Triton. The plates were dried at 37°C for 1 hr, covered with a plate sealer, and stored frozen with desiccant in Ziploc® bags at −30°C until use.

To perform the assay, all the reagents were allowed to come to room temperature before use, except the TMB substrate, which was placed in the 25°C incubator for up to 8 hours until use, and the goat anti-human IgG peroxidase, which was kept at −20°C until use. Specimens, controls, and CAL were diluted 1∶101 in milk buffer and 100 µL of the diluted specimens or controls were added to their respective well and incubated for 1 hr at 37°C. Plates were washed four times with 300 µl of PBS-Triton buffer (wash buffer), and then 200 µL/well of dissociation buffer (citrate buffer, pH 3.0, TEKnova, Hollister, CA) was added and incubated for 15 min at 37°C. The plates were washed and 100 µL of a 1∶5000 dilution of goat-antihuman IgG peroxidase (KPL, Gaithersburg, MD) in milk buffer was added to each well and incubated for 30 min at 37°C. The plates were washed again and incubated with 100 µL/well of TMB substrate (KPL) for 15 min at 25°C. The reaction was stopped by the addition of 100 µL/well of 1 N H_2_SO_4_ (Sigma Aldrich) and the optical density (OD) was read at 450 nm. Normalized OD (ODn) values were calculated for each specimen using a CAL specimen tested on the same plate as follows: ODn = specimen OD/median CAL OD. All controls and CAL specimens were tested in triplicate, diluted individually, and median values were used. All other specimens were tested in singlet but on two separate runs to generate duplicate values and to account for inter-run variations. Mean ODn values of two runs were used for further analysis.

### Two-well avidity-index EIA (AI-EIA)

Micro-well plates were coated with 0.125 µg/ml of purified rIDR-M, using the same coating steps as the one-well assay. HIV-1 positive specimens and controls, diluted 1∶101 in milk buffer, were incubated in duplicate wells for 1 hr at 37°C. After the antibody was bound, the plates were washed four times with 300 µl wash buffer, and then one well was incubated for 15 minutes with 0.1 M dissociation buffer (citrate buffer, pH 3.0, treated well), while the other well was incubated with wash buffer (control well). The plates were washed and 100 µL of a 1∶5000 dilution of goat-antihuman IgG peroxidase (KPL, Gaithersburg, MD) in milk buffer was added to each well and incubated for 30 min at 37°C. The plates were washed again and incubated with 100 µL/well of TMB substrate (KPL) for 15 min at 25°C. The reaction was stopped by the addition of 100 µL/well of 1 N H_2_SO_4_ (Sigma Aldrich) and the optical density (OD) was read at 450 nm. The AI for each specimen was calculated as = (OD of treated well/OD of control well)×100. All specimens were tested on two separate runs to generate two values and the mean AI was used for further data analysis.

### HIV-1 BED Incidence EIA (BED assay)

The assay has been previously described in detail [6], [13] and was performed according to the manufacturer's instructions. The data for specimens used in this study were generated earlier and represent a subset of specimens reported [22].

### HIV Serology

Genetic Systems HIV-1-2 Plus O EIA (Bio-Rad Laboratories, Hercules, CA) was performed according to the manufacturer's instructions. The Cambridge Biotech HIV-1 Western Blot (Maxim Biomedical, Rockville, MD) was performed as per kit instructions.

### Determination of the mean duration of recency

The mean duration of recency was derived from longitudinal specimens of seroconverting individuals representing 3 different HIV-1 subtypes and also for overall for all seroconverters combined. The statistical method used to calculate the mean duration of recency has been described recently [22]. Here, a similar but a more general approach was followed. In brief, a nonparametric survival analysis method for interval-censored data was used to estimate the recency period of the assay. This approach required fewer assumptions than other approaches. It was adapted in our context where it was known that seroconversion had occurred between two time points, t_1_ and t_2_, and assay threshold was crossed between time points, t_3_ and t_4_. Therefore the recency period lies in the interval (t_3_ - t_2_) and (t_4_ - t_1_). If the threshold was not crossed by the last observation then the upper end of this interval was set as censored. These two limits were used to calculate the maximum likelihood estimate of the survival curve. The mean and median estimates of the recency period were directly derived from the survival curve. For the mean to be defined finitely, it was assumed that the event occurred for the longest observed subject at the latest observed time. We employed a SAS macro called EMICM to estimate the survival curve for the recency period [28]. Confidence intervals were estimated based on bootstrap techniques. Upper and lower limits of the interval were derived as 97.5^th^ and 2.5^th^ percentiles of the empirical distribution. We used various threshold values and derived estimates for each of the subtypes and overall recency period.

## Results

### LAg-Avidity EIA Controls

Selected bulk volume HIV-1 positive specimens with OD in the dynamic range were chosen as low positive control (LPC), calibrator (CAL), and high positive control (HPC). The controls and CAL were undiluted plasma specimens. CAL was chosen such that its OD value was between 0.6–0.8, with HPC and LPC bracketing the CAL. A summary of their raw OD values and normalized OD values (ODn) from 94 plate runs from three different plate lots are shown in Table 1. By definition, CAL has an ODn of 1.0 while LPC and HPC were selected to have ODn of approximately 0.5 and 1.5–1.6, respectively. Coefficient of variation for LPC, CAL and HPC were >20% for raw OD values (Table 1) but decreased to <10% for ODn values, demonstrating that normalization of OD value for each specimen is important for reducing inter-run variations.

**Table 1:** Mean OD and ODn values for CAL and controls, including standard deviation (SD) and coefficient of variation (%CV) from 94 runs.

Control ID	Raw OD values		ODn Values	
	Mean OD+SD	%CV	Mean ODn+SD	%CV
**NC**	0.089±0.016	17.5	0.151±0.044	29.5
**CAL**	0.624±0.151	24.3	1.000	0.0
**LPC**	0.305±0.067	21.9	0.495±0.047	9.5
**HPC**	0.996±0.223	22.4	1.609±0.124	7.7

NC-negative control, CAL = calibrator, LPC = low positive control and HPC = high positive control specimens.

### Kinetics of antibody avidity development among different subtypes

**Figure 1:**
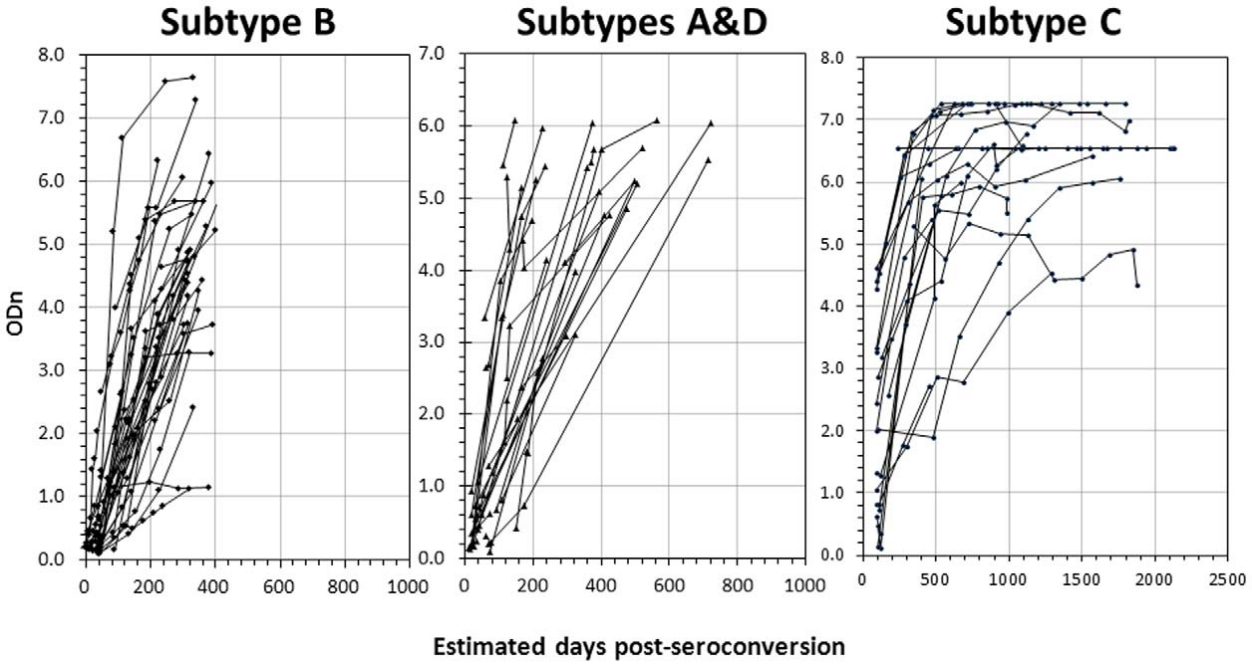
Antibody avidity maturation post-seroconversion as measured by LAg-Avidity EIA in individuals from 4 cohorts representing different geographic locations and subtype infections: Amsterdam and Trinidad cohorts (subtype B; left), Kenya cohort (subtypes A and D; middle), and Ethiopia cohort (subtype C; right). Each line represents a single individual with sequential specimens collected over time post-seroconversion (X-axis; note that scale is different in panel C due to longer follow up).

LAg-Avidity EIA results of specimens from 4 longitudinal cohorts, representing 3 different subtypes, are shown in *Figure 1*. Antibody avidity, as measured by LAg-Avidity EIA, increased in almost all cases over time. Specimens from individuals infected with subtype B (Amsterdam and Trinidad cohorts) were more uniformly spaced with frequent and regular sampling, thus providing a coherent picture of avidity kinetics. In contrast, some individuals infected with subtype A or D (Kenya cohort) or subtype C (Ethiopia cohort) had large inter-sample interval with an irregular sample collection schedule, which was reflected in more heterogeneity of kinetics. It should be noted that the scale is different in the last panel (subtype C) because of longer follow up of these individuals.

### Comparison of BED, LAg-Avidity EIA and Avidity-Index EIA in Seroconverters

**Figure 2:**
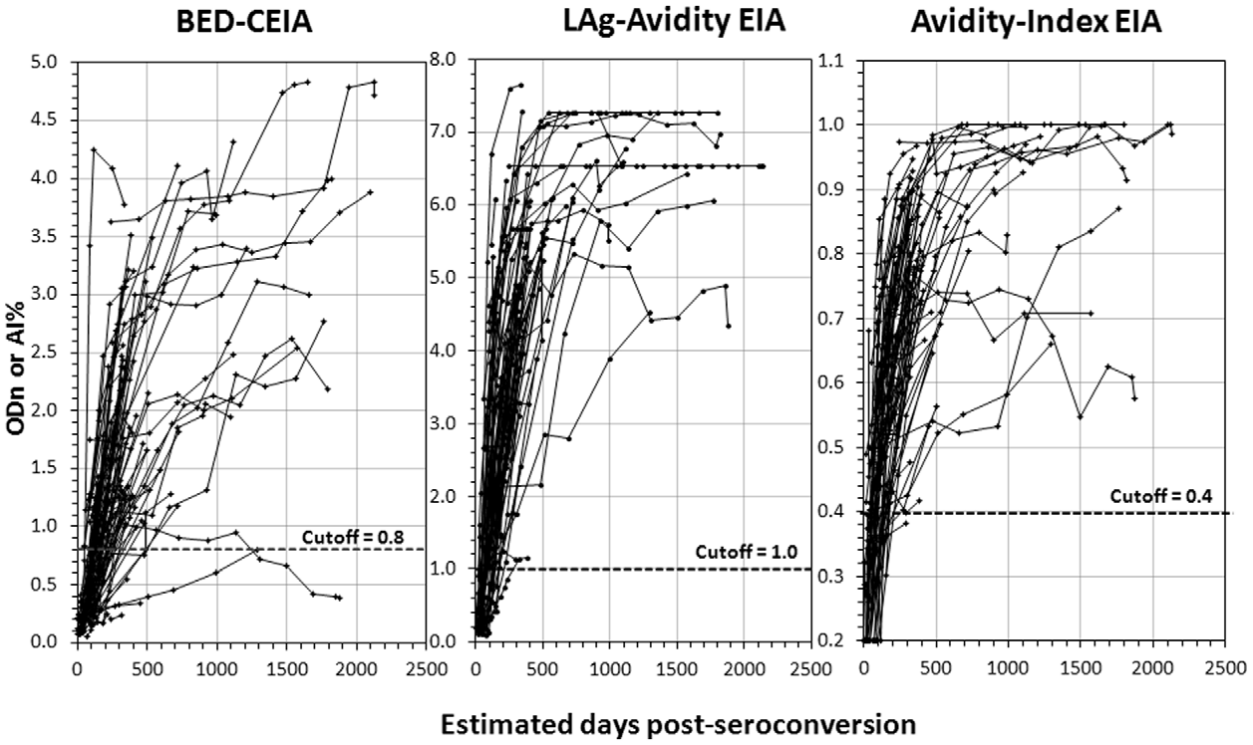
Comparative HIV antibody kinetics following seroconversion as detected by the BED assay (left), LAg-Avidity EIA (middle), and Avidity Index-EIA (right) among 89 seroconverting individuals (subtypes A ,B, C & D). The BED assay measures increasing proportion of HIV-specific IgG in total IgG, while LAg-Avidity EIA and AI-EIA measure increasing avidity of HIV antibody. Both LAg-Avidity EIA and AI-EIA use multi-subtype gp41 recombinant protein, rIDR-M, and pH 3.0 dissociation buffer to distinguish recent and long-term HIV infections.

Compiled antibody kinetics data for all 89 seroconverters for BED, LAg-Avidity EIA and two-well rIDR-M AI-EIA are shown in *Figure 2*. All plots have the same scales on the X-axes for ease of comparison. For the BED assay, most individuals show an increase in HIV antibody levels over time. However, there is more heterogeneity among individual responses, including some which ramp up slowly or even decline over time and go below the cutoff of 0.8 ODn, contributing to the misclassification as recent infection. In contrast, both one-well and two-well avidity assay kinetics were rapid, with less variation among individuals compared to the BED assay. Interestingly, both one-well LAg-Avidity EIA and two-well AI-EIA had similar performance, and none of the individuals tested stayed below the proposed cutoff of 1.0 ODn for LAg-Avidity EIA or the matching cutoff of 40% avidity index for AI-EIA. All subsequent work described here is focused on LAg-Avidity EIA.

### Mean Duration of Recency

**Figure 3:**
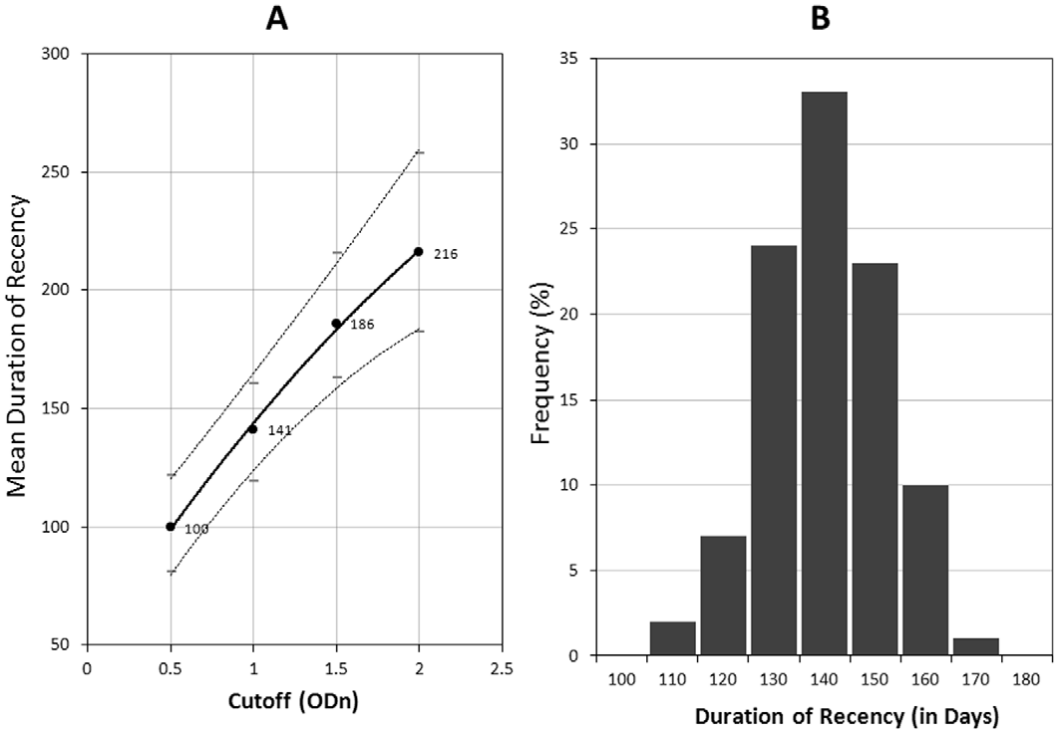
A) Increase in duration of mean recency (solid line) of LAg-Avidity EIA with varying cutoffs from 0.5 to 2.0 ODn. The 95% confidence interval is shown by dotted lines. The numbers shown represent mean duration of recency, ω, at each cutoff. B) Empirical frequency distribution of duration of recency at cutoff of 1.0 for LAg-Avidity EIA.

Mean duration of recency (ω) at varying cutoff for LAg-Avidity EIA was calculated using the non-parametric survival method for interval estimation described earlier [22]. Figure 3A shows the relationship between cutoff value and ω, where the value of ω was 100 days (95% CI 81–122) at 0.5 ODn cutoff and increased to 216 days (95% CI 182–258) at cutoff of 2.0 ODn. At cutoff of 1.0 ODn, the ω value was 141 days (95% CI 119–160). Further analysis was performed using an assay cutoff of 1.0 ODn which should have the least variability due to normalization. The empirical frequency distribution of duration of recency is shown in Figure 3B, which demonstrates that ω has approximately normal distribution with a peak at about 140 days.

Subtype specific analysis, summarized in Table 2, demonstrated that the ω was very similar across the different subtypes and ranged from 132 days (subtype B) to 143 days (subtype C), with significant overlap of confidence intervals (all p-values for pair-wise tests exceed 0.5). This suggests that the LAg-Avidity EIA has similar performance in these cohorts representing 4 different HIV-1 subtypes.

**Table 2:** Summary of mean recency period (in days) for LAg-Avidity EIA at cutoff of 1.0 by cohort and prevalent HIV-1 subtype(s).

Cohort	No. of Subjects (No. of specimens)	HIV-1 Subtypes	Mean Recency Period(95% CI), in Days
Amsterdam & Trinidad	32 (170)	B	132 (104–157)
Ethiopia	23 (143)	C	139 (106–178)
Kenya	34 (80)	A, D	143 (103–188)
**ALL**	**89 (393)**	**A, B, C and D**	**141 (119–160)**

### Misclassification among people with AIDS

Overall, the BED assay misclassified 14 (2.9%; 95% CI 1.4%–4.4%) of 488 individuals with AIDS (with or without TB) as having recent infections (Table 3). The misclassification varied from 0.8% (95% CI 0.0%–2.23%) in Thailand specimens to 4.2% (95% CI 1.8%–6.6%) in HERS specimens. In comparison, misclassification with LAg-Avidity EIA was 0.2% (95% CI 0.0%–0.6%) with only 1 of the total 488 individuals being misclassified as having recent infection.

**Table 3:** Frequency of false recent classification and false recent rates (FRR) for the BED and LAg-Avidity EIA in specimens collected from individuals with AIDS and AIDS with TB co-infections.

	Frequency of False Recent Classification (%)	
Cohort	BED-CEIA	LAg-Avidity EIA
US, AIDS (n = 261)	11 (4.2%)	0 (0.0%)
Thailand, AIDS+TB (n = 128)	1 (0.8%)	0 (0.0%)
Cote d'Ivoire , AIDS+TB (n = 99)	2 (2.0%)	1 (1.0%)
**OVERALL (n = 488)**	**14 (2.9%)**	**1 (0.2%)**

### Application to a cross-sectional specimen set

**Figure 4:**
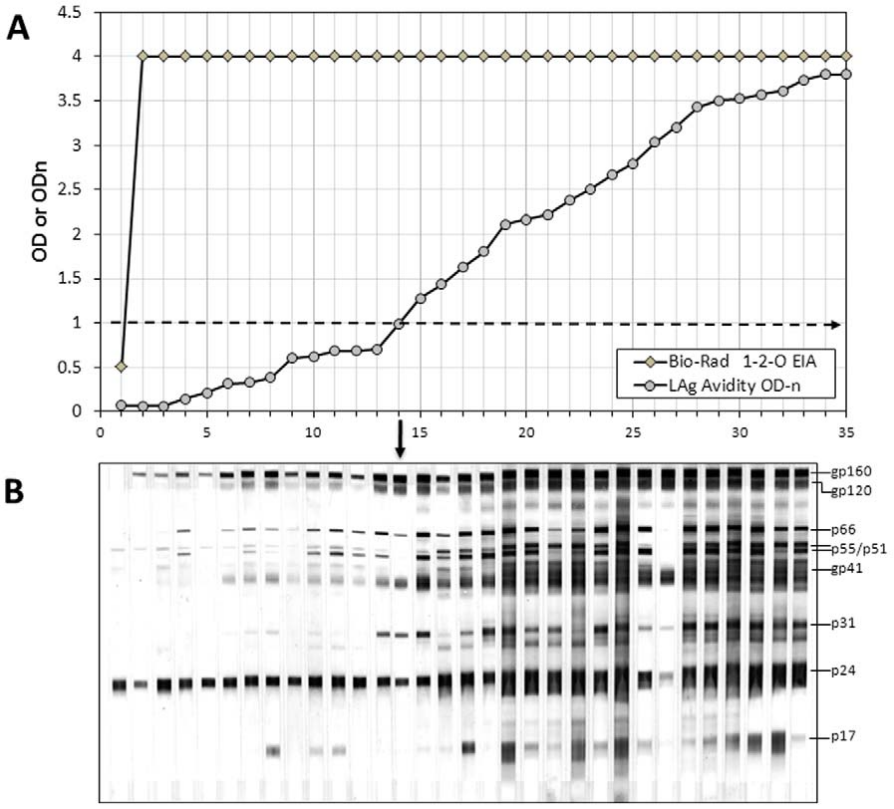
A) Comparative OD or ODn values of a cross-sectional specimen set tested with Genetics Systems HIV-1/HIV-2 Plus O EIA and LAg-Avidity EIA. Specimens are arranged in ascending ODn on LAg-Avidity EIA to represent avidity maturation since seroconversion; a horizintal line at 1.0 indicates cut-off for recent and long-term classification. Only first 35 specimens are shown for clarity. B) Corresponding HIV-1 Western blot banding profile of first 32 specimens showing increase in number and intensity of bands typical of antibody maturation. For the purpose of incidence estimation, vertical arrow indicates cutoff; recent infections = left of the arrow, long-term infections = right of the arrow.

As a proof of concept, we applied this assay to 97 HIV-1 positive specimens purchased from a commercial source (see Methods). These specimens can be considered from a cross-sectional specimen set and all, except one, were strongly positive on diagnostic Bio-Rad HIV-1-2-O assay with OD value of 4.0 (the upper limit of the reader). One specimen had a low OD value of 0. 510 but was repeatedly positive (above the cutoff) on the diagnostic EIA (Figure 4A). The specimens had varying avidity as measured by LAg-Avidity EIA with 14 specimens being classified as recent infections (ODn≤1.0). The specimens were arranged in ascending order of avidity to represent imputed increasing time from seroconversion (Figure 4A) and were tested with the HIV-1 Western blot assay (Figure 4B). For simplicity, results of only the first 35 individuals with the lowest LAg-Avidity assay reactivity are shown (panel A), along with corresponding Western blot results for the first 32 individuals (panel B). Although these are single specimens from different individuals, when arranged in the order of increasing avidity, the WB banding pattern showed increased number and intensity of bands representing maturation of HIV antibodies that are typical of a seroconverting individual. The first 14 specimens are classified as recent by the LAg-Avidity EIA (demarcation shown by an arrow) and have weaker/partial banding patterns. Subsequent specimens are classified as long-term infection with only 19 shown here for simplicity. The WB banding patterns were typical of long-term infections with strong antibodies to most HIV proteins. All the rest had full WB banding patterns. Of particular interest is specimen ID 27 that has strong antibodies to envelope proteins but weak anti-gag (p24) antibodies, typical of individuals with advanced disease. Specimens such as these are more often misclassified as recent with the BED assay, but they are correctly identified as long-term infections with the LAg-Avidity EIA.

## Discussion

Intensive efforts by national HIV control programs of many countries, with support from several global initiatives, such as PEPFAR and the Global Fund to Fight AIDs, TB, and Malaria, have made significant impact on transmission of HIV infection [16], [29]. UNAIDS recently reported >25% reduction in HIV incidence in 33 countries [30], with 22 of them in sub-Saharan Africa, and HIV incidence appears to have stabilized or is showing signs of decline in 5 of the most affected countries. However, the model-based incidence estimates are retrospective and not as timely as needed for targeting prevention efforts and allocation of resources. Moreover, the estimates depend on several assumptions and do not lend themselves to sub-population estimates and risk-factor analyses that are so crucial for targeting interventions. Therefore, laboratory-based methods applied to cross-sectional samples continue to remain attractive, with the potential to provide information on current transmission dynamics and hot-spots [2].

We previously described development of a novel single-well avidity assay (LAg-Avidity EIA) and a two-well avidity index assay (AI-EIA), both using a multi-subtype recombinant gp41 protein (rIDR-M), to detect recent HIV-1 infections in cross-sectional specimen sets [25]. We extend this study to demonstrate that the LAg-Avidity EIA can detect and distinguish recent and long-term infections with high accuracy and has similar performance in 4 divergent subtypes tested. The novel features of LAg-Avidity EIA are: 1) the ability to measure antibody avidity in a single well simply by limiting the amount of antigen available to bind the antibodies, 2) the use of a multi-subtype gp41 recombinant protein, which reduces or eliminates bias among different subtypes or population, resulting in similar duration of recency, 3) the use of a low pH buffer (pH 3.0) to facilitate the differentiation between recent and long-term infection, and 4) the improved reproducibility and increased number of specimens that can be tested per plate due to the single well format for measuring antibody avidity. Avidity maturation kinetics were very similar by both 1-well LAg-Avidity EIA and 2-well AI-EIA, indicating that this new approach measures antibody avidity but in a single-well (Figure 2).

Currently, the BED assay is the only assay commercially available specifically to measure HIV-1 incidence in cross-sectional populations and is used in more than 50 laboratories worldwide [18], [31], [32], [33]. The assay has high precision [34], although its accuracy to detect recent infection has been questioned due to false recent classification resulting in elevated incidence estimates [31], [35], [36], [37]. Moreover, recent analysis demonstrated that the assay has a significantly higher mean duration of recency in African populations than in other populations [22]. Both these limitations appear to result from the capture format of the assay, which captures both HIV-1 IgG and non-HIV-1 IgG, to determine the proportion of HIV-1 IgG; elevated total IgG levels in African populations contribute to this bias [22].

Development of a new generation of avidity assays, described in our recent work [25], was geared to address these limitations and those that may impact modified commercial assays. We developed a multi-subtype gp41 recombinant protein and used it in two different avidity assays, including the novel single-well format. We also describe development of control specimens, including CAL which is important for normalization of OD values to reduce inter-run variations and determination of the assay cutoff. The HPC and LPC were developed to bracket the CAL in the dynamic range. Monitoring values of controls over >90 plates demonstrated that calculation of ODn improves consistency and reduces coefficient of variation to <10%, especially in the dynamic range that is important for classification of recent and long-term infection (Table 1).

We demonstrate here that the LAg-Avidity EIA has a similar mean duration of recency among 2 cohorts from Africa (Kenya, representing subtype A &D, and Ethiopia, representing subtype C) and 2 additional cohorts from Amsterdam and Trinidad, representing subtype B (Table 2). Overall, the mean duration of recency or ω increased from 100 days at a cutoff of 0.5 ODn to 216 days at 2.0 ODn (Figure 3A). A cutoff of 1.0 ODn corresponds to ω of 141 days (95% CI 119–160) and was chosen for application in cross-sectional settings because the CV is the lowest close to the CAL specimen and the duration of recency period is between 4 and 5 months, which is sufficiently long to detect new infections for good incidence estimates. Subtype-specific analysis show that window periods are similar and range from 131 to 143 days (Table 2). Two subtypes, A & D from Kenya, were analyzed together due to a limited number of samples available to perform statistically sound calculation of ω for each subtype. However, the pattern of avidity kinetics were very tight, irrespective of HIV-1 subtypes (Figure 1 & 2), with uniform distribution of recency periods (Figure 3B), indicating that ω values for 4 subtypes are quite similar. Similar analysis of the BED window period recently showed a non-normal distribution with a right tail, although these results were derived from much larger data set [22]. Additional work with more longitudinal specimens, representing different populations and subtypes, will be required to further refine our findings. However, it is important to note that ω values are also affected by precision of seroconversion time as determined by collection interval between last negative and first positive samples and frequency of sample collection from seroconverting individuals. Therefore, it is expected that ω will be revised as more seroconverters are tested as part of ongoing calibration of new incidence assays by us and other laboratories as the assay becomes widely available.

It was very encouraging that the misclassification rate among 488 individuals with AIDS was only 0.2% (1 of 488) for the LAg-Avidity EIA, compared to 2.9% (14 of 488) with the BED assay. Additional testing of specimens from two different ART-naïve populations with documented long-term infections (>1 year) also suggest low false recent classification rate (FRR) (data to be published separately), which is consistent with characteristics of an acceptable assay to provide accurate incidence estimates [38]. This suggests that the LAg-Avidity assay is likely to perform better among those with long-term infections, including AIDS; however, ongoing studies are needed in different populations, HIV-1 clades, and state of the epidemic before this determination can be made definitively.

Both less-sensitive EIAs and the BED assay show increased “misclassification” when individuals are on anti-retroviral treatment (ART) or are elite suppressors with low viral load [39], [40], [41]. With expanded treatment programs in most countries, this could have significant impact on incidence estimates unless that information is collected during the survey. In a limited study of individuals on ART, the LAg-Avidity EIA performed better than the BED assay (unpublished data); however, a larger sample size and information on duration of treatment are needed before we can quantify the impact of ART. If misclassification among people on ART continues to be a significant factor by antibody-based assays, use of another assay based on a different principle or detection of ARV in samples classified as recent should be considered which can improve the incidence estimate [17]. It is to be noted that the latter exercise needs to be conducted only on samples that are classified as recent, limiting the cost of additional testing for ARV.

As in diagnostic assays, two or more tests based on different principles (e.g., BED and avidity assay) can be used in an algorithm (parallel or serial) to increase the predictive value of detecting recent seroconversion and to exclude false recent misclassifications. In addition, further testing for CD4, viral load and ARV have been proposed to improve the estimates. However, there must be demonstrated contribution and advantages of each of the steps/assays for improved incidence estimates. Otherwise, increased cost of performing multiple assays will not be worthwhile. Additional testing of cross-sectional specimen sets with the BED and LAg-Avidity assays, as well as other incidence assays, will help in formulating a definitive guidance about the utility of multi-assay algorithm, if any.

Often, and incorrectly so, the incidence assays are characterized for sensitivity and specificity to detect recent HIV infection. Although long-term specificity of the assay is important among those infected >1 year, sensitivity and specificity are not relevant for detecting and distinguishing recent from long-term infections during the early phase of infection since the mean duration of recency is applicable only to populations, not to individuals. Accordingly, the mean recency period of 141 days is meant for incidence estimate calculation only. However, data from the frequency distribution of recency period (Figure 3B) indicate that those below cutoff of 1.0 are very likely to have seroconverted within the last 180 days with high probability; hence, such data may be useful for research and public health applications at the individual subject level, specifically for the prevention purposes, transmission of drug-resistance viruses and similar studies.

With development of controls, CAL and other key reagents, this novel LAg-Avidity assay is now well-standardized. Our application in a cross-sectional specimen set suggest that the assay detects recent HIV-1 infection with high accuracy and that an increase in ODn reflects avidity maturation of HIV antibodies following seroconversion, as confirmed by evolving Western blot banding pattern (Figure 4). As part of the World Health Organization (WHO) Incidence Working Group, we are planning additional independent multi-center evaluations of the LAg-Avidity EIA for detecting recent infections.

HIV vaccine developmental work has lately focused on elicitation of high avidity antibody as a marker for effective humoral response and possibly as a surrogate for protection [42], [43]. Multiple versions of avidity assays are in use to evaluate the vaccine response; however these avidity assays are difficult to compare or to transfer from one laboratory to another due to lack of standardization and appropriate quality controls. Moreover, most assays use antigens derived from subtype B or those used for a specific candidate vaccine. The standardized avidity assay described here may also have applications for studying antibody maturation in vaccine recipients if gp41 antibodies are elicited, irrespective of the subtype-specific vaccine. This assay can also be used to evaluate antibody maturation in HIV-infected individuals following break-through infections. This new concept of simply limiting the antigen to measure antibody avidity, all in a single well, can also be extended to other antigens and pathogens where 2-well avidity index based assays are routinely used.

We realize that in-house incidence assays will not be used widely unless they are produced and are available commercially, as has been the limitation for some of the other in-house assays described earlier [11], [44]. Therefore, development of a commercial kit is an important step towards wider availability and application. Recently, we have transferred the technology to commercial companies and expect availability of a kit(s) in the near future which will further facilitate additional validation and use of the test.

In summary, the need for a reliable laboratory method to estimate HIV-1 incidence from cross-sectional specimen sets cannot be overemphasized. Such a tool can help maximize impact of our efforts to reduce new HIV infections by zeroing in on hot-spots and permitting the best use of scarce resources. This novel single-well avidity assay has performance characteristic suitable for detecting recent HIV-1 infection with high accuracy in divergent subtypes or populations and should provide a reliable laboratory tool to estimate HIV-1 incidence worldwide.
